# Mechanisms of Oncolysis by Paramyxovirus Sendai

**Published:** 2015

**Authors:** O. V. Matveeva, G. V. Kochneva, S. V. Netesov, S. B. Onikienko, P. M. Chumakov

**Affiliations:** Biopolymer Design, 23 Nylander Way, Acton, Massachusetts, United States; Center of Virology and Biotechnology “Vector”, Koltsovo, Novosibirsk Region, Russia; Novosibirsk State University, Novosibirsk, Russia; Department of Military Field Therapy, Kirov Military Medical Academy, St. Petersburg, Russia; Engelhardt Institute of Molecular Biology, Moscow, Russia

**Keywords:** attenuated measles virus strains, Newcastle disease virus, Sendai virus, oncolytic paramyxoviruses, viral anti-tumor mechanism, viral anticancer immune stimulation, cancer therapy

## Abstract

Some viral strains of the Paramyxoviridae family may be used as anti-tumor agents. Oncolytic paramyxoviruses include attenuated strains of the measles virus, Newcastle disease virus, and Sendai virus. These viral strains, and the Sendai virus in particular, can preferentially induce the death of malignant, rather than normal, cells. The death of cancer cells results from both direct killing by the virus and through virus-induced activation of anticancer immunity. Sialic-acid-containing glycoproteins that are overexpressed in cancer cells serve as receptors for some oncolytic paramyxoviruses and ensure preferential interaction of paramyxoviruses with malignant cells. Frequent genetic defects in interferon and apoptotic response systems that are common to cancer cells ensure better susceptibility of malignant cells to viruses. The Sendai virus as a Paramyxovirus is capable of inducing the formation of syncytia, multinuclear cell structures which promote viral infection spread within a tumor without virus exposure to host neutralizing antibodies. As a result, the Sendai virus can cause mass killing of malignant cells and tumor destruction. Oncolytic paramyxoviruses can also promote the immune-mediated elimination of malignant cells. In particular, they are powerful inducers of interferon and other cytokynes promoting antitumor activity of various cell components of the immune response, such as dendritic and natural killer cells, as well as cytotoxic T lymphocytes. Taken together these mechanisms explain the impressive oncolytic activity of paramyxoviruses that hold promise as future, efficient anticancer therapeutics.

## INTRODUCTION

The existing approaches to the treatment of metastatic cancer are often ineffective. Therefore, new antitumor agents and new methods for the destruction of tumor cells are required. The idea of using viruses to treat malignant diseases is not a novel one. It dates back to the beginning of the XX century, when spontaneous regression of tumors was first reported in some patients after viral infection or vaccination with a live virus. The first reviews discussing this issue were published in the 1950s [1-3]. Viruses capable of specific destruction of malignant cells without affecting normal cells were later referred to as oncolytic. Specific destruction of cancer cells is caused by selective replication of a virus in these cells and virus-induced activation of anticancer immunity.

Various viruses with both DNA and RNA genomes possess oncolytic activity. The genomic DNA of such viruses may be single-stranded, e.g. in parvoviruses [4], or double stranded, e.g. in oncolytic adenoviruses [5] and poxviruses [6]. The genomic RNA of oncolytic viruses can also have different forms: positive sense single- stranded RNA (enteroviruses [7]), double stranded RNA (reoviruses [8]), or negative sense single-stranded RNA (paramyxoviruses and rhabdoviruses in [9]).

Some members of the Paramyxoviridae family, including a number of attenuated vaccine strains of the measles virus [10], various animal viruses that are non-pathogenic for humans, such as Newcastle disease virus [11-13] and Sendai virus (to which this review is dedicated), have been studied as potential anticancer agents.

## ANTITUMOR ACTIVITY OF THE SENDAI VIRUS

**Studies of the Sendai virus and its oncolytic properties**

**Fig. 1:**
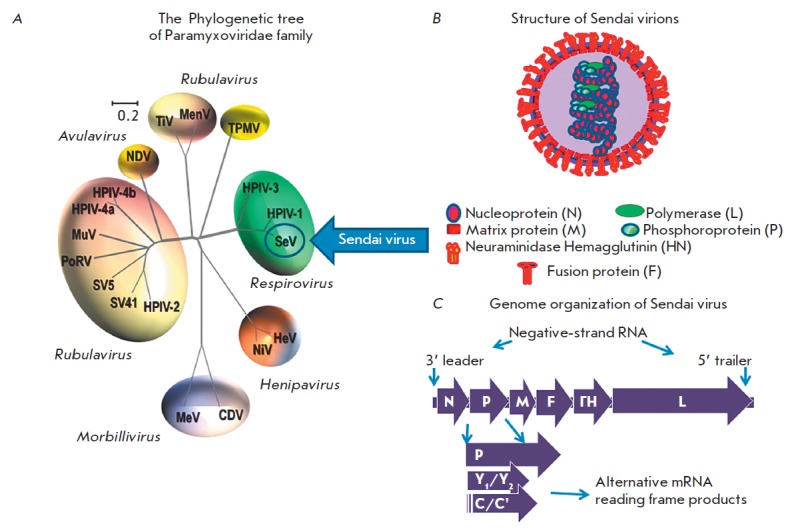
Paramyxoviridae phylogenetic tree along with a virion composition and genomic organization scheme of the Sendai virus. A) The phylogenetic tree based on the alignment of the amino-acid sequences of the HN genes of selected* Paramyxoviridae *subfamily members. The family members with proven oncolytic properties are circled. The tree was generated by Clustal W multiple alignments using the Neighbor-Joining method. Viruses are grouped according to genus and abbreviated as follows. *Morbillivirus *genus: MV (measles virus), CDV (canine distemper virus); *Henipavirus* genus: HeV (Hendra virus), NiV (Nipah virus); *Respirovirus *genus: SeV (Sendai virus), hPIV3 (human parainfluenza virus 3); *Avulavirus *genus: NDV (Newcastle Disease Virus); *Rubulavirus *genus: hPIV2 (human parainfluenza virus 2), hPIV-4a (human parainfluenza virus 4a), hPIV-4b (human parainfluenza virus 4b), MuV (mumps virus), PoRV (porcine rubulavirus), SV5 (simian parainfluenza virus 5), SV41 (simian parainfluenza virus 41); TiV (tioman virus); MenV (menangle virus); Unclassified: TPMV (Tupaia paramyxovirus), B) Structure and composition of virion, C) Genomic organization of the Sendai virus

The oncolytic properties of the Sendai virus, which is also known as a murine parainfluenza virus type 1 or the hemagglutinating virus of Japan, have been studied particularly within the last 10 years. This paramyxovirus belongs to genus *Respirovirus *of the Paramyxoviridae family. *Fig. 1* shows a phylogenetic tree of the family Paramyxoviridae (A), the structure of the Sendai virus virion (B), and the structure of its genome (C). The Sendai virus genome is a negative sense single- stranded RNA, which is 15.3 kb long and contains six protein-encoding genes. Two of these genes encode the surface glycoproteins HN and F; three encode the nucleocapsid proteins NP, P, and L; and the last one encodes the non-glycosylated internal matrix protein M. A distinctive feature of paramyxoviruses is the presence of an F protein, which promotes membrane fusion at neutral pH. The F protein is synthesized as an inactive precursor protein, the F0 protein, which is subsequently cleaved by cellular proteases into two subunits, F1 and F2, which remain linked to each other via disulfide bridges [14].

In nature, the arginine-specific serine protease “Clara” is most likely responsible for the maturation of the virus [15-7]. The ability to process the F0 protein defines the tissue tropism of paramyxoviruses [18]. Only inactive precursor virus particles can form in the absence of proteolytic activation of F0 [19]. When the Sendai virus is grown for research purposes in cells which do not produce the protease required for the activation, this enzyme (e.g., trypsin) must be added to the extracellular environment.

The Sendai virus causes easily transmitted respiratory tract infections in mice, hamsters, guinea pigs, rats, and sometimes in pigs [20]. The Sendai virus can spread both through the air and through direct contact. It can be found in mice colonies around the world but is believed to be completely safe for humans [20]. In the USA, the Sendai virus is approved for clinical trials aimed at immunization against diseases caused by the parainfluenza type 1 virus in children. This research is based on the assumption that the Sendai virus and parainfluenza virus 1 induce production of cross-reactive antibodies. It was found that intranasal administration of the Sendai virus is well tolerated and it induces the production of antibodies that can neutralize parainfluenza virus 1 [21]. This study is important as proof of the Sendai virus’ safety for humans.

A number of studies conducted in Japan demonstrated that the attenuated virus, genetically modified to be non-pathogenic for rodents, can spread rapidly in tumor cells and destroy them without affecting the surrounding normal cells. This property often leads to tumor growth suppression in mice. The list of tested xenotrasplanted human tumor models includes fibrosarcoma cells, pancreas epithelioid carcinoma, and colon cancer [22]. The use of a recombinant Sendai virus has resulted in significant suppression of tumor growth in mouse models and even in complete eradication of mature brain tumors [23]. Similar results were obtained for xenotransplantation of human sarcoma and prostate cancer cells into mice [24, 25]. The recombinant Sendai virus has been shown to be highly efficient in destroying melanoma, hepatocellular carcinoma, neuroblastoma, squamous cell carcinoma, and human prostate cancer in rat xenograft models [26]. It has been demonstrated that even after inactivation by ultraviolet light (UV), Sendai virus preparations are effective against colon [27, 28], bladder [29], and kidney [30] cancer in syngeneic mice. The efficiency of UV-inactivated Sendai virus has also been demonstrated for murine xenografts of human prostate cancer [31]. In all these studies, Sendai virus therapy led to complete tumor regression or major suppression of its growth.

In 1964, a short-term remission following intravenous administration of live Sendai virus was reported in the United States in a patient with acute leukemia [32].

**Studies of the oncolytic properties of the Sendai virus in Russia**

In the mid-1950s, Academician of the Academy of Medical Sciences V.M. Zhdanov obtained a Sendai virus strain from Japan; the strain was later used for research purposes as a model pathogen at the D.I. Ivanovski Institute of Virology. At the end of the 1960s, the strain was transferred from the lab of V.M. Zhdanov to V.M. Senin (RCRC RAMS) and underwent about 30 passages in chicken embryos. The fact that the Sendai virus is non-pathogenic for humans makes it a promising therapeutic agent against malignant diseases. In the early and mid-1990s, V.M. Senin and his colleagues tested the strain of Sendai virus on volunteers, patients in Moscow and St. Petersburg hospitals, with various malignant grade III and IV diseases. Although in some patients improvement was transient or not observed at all, other patients achieved long-term remissions, even in the cases where tumors had been previously considered inoperable and the virus was used as a monotherapy. In these cases, resorption of primary tumors and metastases was observed and all objective and subjective signs of cancer disappeared. In some cases, after one or two courses of Sendai virus therapy no signs of the disease were discovered even within 5-10 years or more. Brief histories of these patients are presented in the text of the patent [33, 34]. The only reported side effect was short-term fever within 24 hours of virus administration.

The Sendai virus strain used in these tests was deposited in the American Type Culture Collection (ATCC) as PTA-13024 and PTA-121432. PTA-13024 contains the virus in frozen allantoic fluid, and PTA- 121432 contains the virus in lyophilized form. The primary nucleotide sequence of the virus strain has been deposited in the database GenBank as KP717417.1.

## MECHANISMS OF ONCOLYSIS BY PARAMYXOVIRUSES

**Direct killing of malignant cells**

**Fig. 2:**
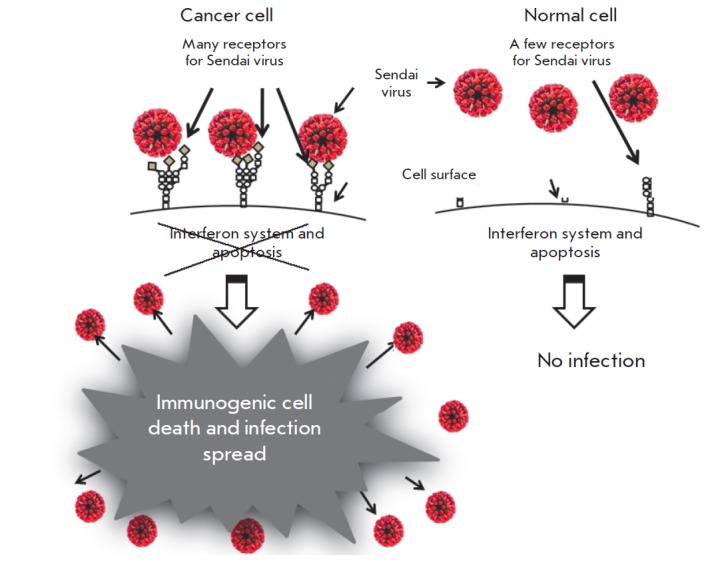
Sendai virus infection and spread in malignant but not in normal cells. First level of virus specificity for cancer cells is related to overexpression of specific receptors for paramyxoviruses. Sialic acids residues in the form of sialoglycoproteins serve as receptors for the Sendai virus. These sialoglycoproteins are frequently overexpressed in malignant cells. Another level of oncoselectivity is related to frequent genetic defects of cancer cells that help viral replication. During the malignant progression cancer cells accumulate many genetic changes. Along with mutations that promote accelerated proliferation and invasion, many cancerous cells lose the abilities to produce interferon and to respond to interferon by induction of the antiviral state. Such abnormalities make these cells highly susceptible to viral infection. Therefore, because cancer cells are overexpressing surface receptors and are commonly defective in antiviral immunity the Sendai virus could easily replicate in malignant cells, but not in normal cells

*Higher affinity of paramyxoviruses for malignant rather than normal cells. *Sialic acid polymers are cellular receptors for some paramyxoviruses [35, 36]. Since a virus binds to its receptor with high affinity, a large number of sialic acid residues on the surface of tumor cells contribute to preferential binding of a virus to malignant, rather than normal cells, which, in turn, leads to a higher concentration of the virus in tumors and metastases compared to normal tissues. *Fig. 2* shows such preferential binding of the Sendai virus to cancer cells.

It has been shown that the viability of human prostate cancer cells, PC3 and DU145, is significantly reduced by a UV-inactivated Sendai virus. Apoptosis has been observed in PC3 cells within 24 hours of treatment with the Sendai virus, with no inhibition of normal prostate epithelium growth [31]. According to the authors, the results of this research confirm that the susceptibility of prostate cancer cells to the Sendai virus can be attributed mostly to a large number of sialylated viral receptors on the surface of the cells and, therefore, to their greater affinity for the virus.

There is also an alternate route for Sendai virus infection of cells which does not involve sialic acid [37]. In this case, the F protein binds to the hepatocyte-specific asialoglycoprotein receptor, ASGR. However, the exact mechanism of this route, as well as its possible role in the oncolytic potential of the virus, requires further investigation.

*Disruption of interferon and apoptosis cell systems. *It is well known that mutations and other genetic alterations accumulate in tumor cells during the progression of the disease, contributing to the disruption in the interferon response system [38, 39]. Moreover, the progression of malignancy unbalances the system responsible for apoptosis [39, 40]. As a result, tumor cells lose their ability to induce the synthesis of interferon, to acquire resistance to viral infections, and to respond to the interferon antiproliferative action. They also lose their ability to progress to apoptosis, despite the signal received. These changes result in tumor progression and growth.

Viruses can replicate by exploiting the same disruptions that promote tumor growth, leading to a larger scale of death among malignant cells compared to normal ones. *Fig. 2* illustrates the differences between malignant and normal cells, which make the infection of cancer cells more likely, more efficient, and results in immunogenic death of malignant cells and further spread of the virus within the tumor.

**Fig. 3:**
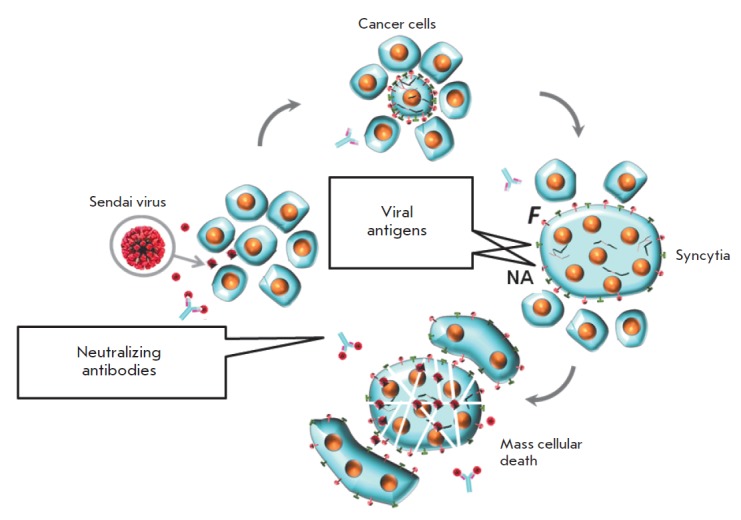
Sendai virus infection may spread through syncytium formation to achieve an accelerated elimination of cancer cells. In natural hosts virus infected cells start expressing the viral fusion protein (F) on the cell surface that forces fusion of infected and surrounding non-infected cells into large polykaryonic structures known as syncytia. The syncytia support viral replication through continuous fusion with neighboring cells, even in the presence of high titers of neutralizing antibodies. Eventually, the syncytia die, which assists in viral oncolysis. The fusion protein of the Sendai virus is synthesized as an inactive precursor (F_0_), and proteolytic cleavage is needed to convert it to active F_1_ that can promote syncytia formation. A tumor-resident host protease is needed for the efficient formation of syncytia

*Formation of malignant cells syncytium. *Some members of paramyxoviruses have developed a mechanism for spreading the infection which involves the fusion of infected and uninfected cells. Such fusion leads to the formation of a syncytium, a large multinucleus structure. Infected cells can fuse with 50–100 neighboring cells to form a syncytium [41]. Infection of new host cells via fusion makes possible the spread of a viral infection without the release of virus particles from the cells. Therefore, the ability to form syncytium represents one of the strategies used by the virus to avoid host-neutralizing antibodies, which otherwise would inactivate it. *Fig. 3* illustrates Sendai-virus-induced syncytium formation. Syncytium typically survives *in vivo *for 4-5 days only and dies afterwards.

It has been suggested that the ability of certain viruses to induce syncytium formation is related to their oncolytic potential. This hypothesis is supported by the fact that it is possible to transfer genes that encode the fusion proteins required for syncytium formation from one type of virus to another. It has been shown that such transfer imparts oncolytic potential to viruses that had not possessed it previously [42, 43]. This potential can be further enhanced by amino acid substitutions, resulting in increased production of proteins capable of cell fusion induction [44, 45]. Even plasmids that encode membrane glycoproteins with a similar function can cause significant tumor regression [46-48].

**Destruction of malignant cells mediated by specific anti-tumor immunity**

*Paramyxoviruses neuraminidase (NA) removes sialic acid from the surface of malignant cells. *It is known that an increased level of sialylation of cell membranes is associated with progression of the malignancy and invasive and metastatic potential of cells [49-54]. It has been demonstrated that certain sialylation inhibitors can reduce the malignancy of cancer cells [55-57].

One of the possible mechanisms linking the increased sialylation with a malignant phenotype is the creation of a thick “coat” on the cell surface that may mask cancer antigens and protect malignant cells from immunosurveillance. Desialyation of tumor cells reduces their growth potential, making them available to natural killer cells (NK). Moreover, sialidase-treated tumor cells better activated NK cells for IFN-γ secretion. It has been shown that the activity and cytotoxicity of NK cells depend on the expression of tumor cell surface-specific sialic acids [58].

Hemagglutinin-neuraminidase (HN) is a protein that can induce hemagglutination and possesses enzymatic activity. Neuraminidase (NA), a subunit of the HN molecule, is an enzyme (sialidase) which cleaves sialic acid from the surface of a cell [35, 36]. NA is encoded and synthesized by certain members of oncolytic paramyxoviruses, including the Newcastle disease virus, Sendai virus, and mumps virus. NA recognizes sialic acid polymers as cell surface receptors [36]. NA also promotes cell fusion, which helps the forming virions to spread within the tissue, avoiding interaction with host antibodies.

**Fig. 4:**
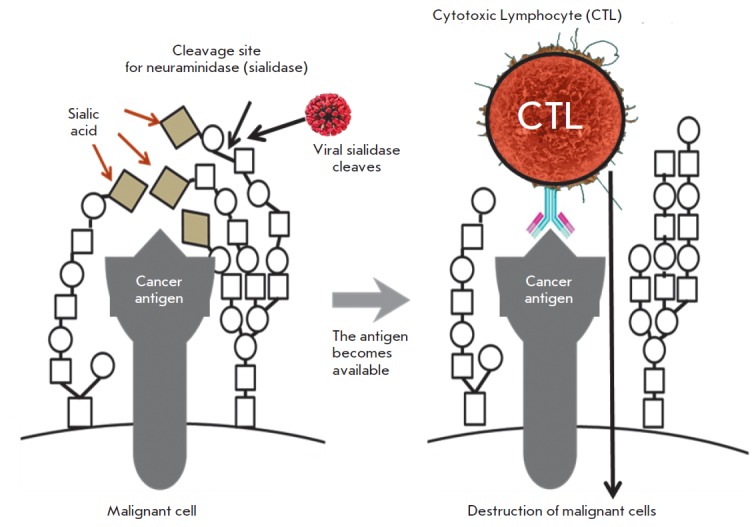
Death of cancer cells through activation of the immune response against cancer cells triggered by the removal of syalic acid residues from the cancer cell’s surface by viral sialidase. Metastatic cancer cells often overexpress sialic acid-rich cell-surface glycoproteins that render a negative charge and electrostatic repulsion between cells that facilitates cancer cells entry into the blood stream, thereby promoting metastasis. One of the possible mechanisms linking increased sialylation with a malignant phenotype is the creation of a thick “coat” on the cell surface that may mask cancer-antigens-protect malignant cells from immunosurveillance. Removing some sialic acid residues by sialidase can unmask cancer-specific antigens and make cells visible to the immune system. The removal of sialic acids from tumor cells is associated with a reduced growth potential, activation of NK cells, and secretion of IFN-gamma. The hemagglutinin- neuraminidase proteins present in the Sendai virus and some other paramyxoviruses possess neuraminidase (sialidase) activity, and, therefore, its action on the surface of cancer cells may dramatically increase the induction of the cytotoxic T-cell response

Removal of sialic acid residues can lead to a significant change in the ability of B lymphoma cells to stimulate cytolytic T lymphocytes. In an experiment with three different types of sialidases, one of which was Newcastle disease virus NA [59], it was found that this enzyme can cleave 2,3-, 2,6- [60], and 2,8-linkages between sialic acid residues [61]. It has also been shown that there are no significant differences in *in vitro* specificity for the cleaved substrate between the Newcastle disease virus, Sendai virus, and the mumps virus [62]. These observations suggest that once a tumor is treated with the virus, malignant cells become desialylated and this fact contributes to enhanced anti-tumor immunesurveillance. *Fig. 4*illustrates a hypothetical process of sialic acid removal from the surface of malignant cells by Sendai virus sialidase, revealing tumor antigens, which subsequently become available for recognition by cytotoxic lymphocytes.

*Stimulation of interferons (IFN) type I and II production.* The Sendai virus acts as a powerful stimulant of interferon α (IFN-α) production in human peripheral blood leukocytes (HPBL) [63]. The virus induces secretion of at least nine different types of IFN-α: 1a, 2b, 4b, 7a, 8b, 10a, 14c, 17b, and 21b. The main one among them is IFN-α1a, which accounts for approximately 30% of the total leukocyte IFN-α [64]. The Sendai virus can also stimulate IFN-γ production in HPBL [65]; therefore, it has been chosen for human leukocyte interferon production on an industrial scale [66].

A UV-inactivated Sendai virus can induce the secretion of IFN-α and IFN-β in certain tumor cell lines [31]. The inactivated virus induces type I IFN secretion by murine dendritic cells. This induction does not depend on cell fusion; however, the F protein is apparently responsible for the effect [67].

It has been shown that stimulation of interferon synthesis promotes oncolytic immune surveillance in several ways. Type I interferons and IFN-γ significantly improve the presentation of the antigens that are dependent on major histocompatibility complex type I (MHC I). IFN-γ also substantially promotes MHC II-dependent antigen presentation. Both of these processes increase the presentation of tumor-specific antigens by malignant- and specific antigen-presenting cells, which promotes the proliferation and activity of anti-tumor cytotoxic T-cells. The interferons can also inhibit angiogenesis by neutralizing angiogenic stimuli coming from the tumor cells and inhibiting the proliferation of endothelial cells. This inhibition is correlated with the lower vascularity of the tumor and subsequent slowing of its growth (see Reviews [68-70]).

*Paramyxovirus stimulates the production of other cytokines*. It has been shown that the Sendai virus can stimulate production of IL-2 [65], macrophage inflammatory protein-1α and -β, and many other cytokines in HPBL [63]. Administration of the Sendai virus to animals demonstrated that both live and UV-inactivated viruses stimulate the secretion of interleukin-6 [27]. It has been determined that the fusion protein (F) of the Sendai virus is responsible for the stimulation of interleukin-6 secretion in dendritic cells [67]. Administration of a UV-inactivated Sendai virus to a patient with a kidney cancer tumor caused the expression of chemokine CXCL10 (also known as protein 10, which can induce interferon-γ) [30].

*Paramyxoviruses can stimulate natural killer (NK) cells. *Activated NK cells can destroy tumor cells without prior antigen stimulation. These cells are part of the important branch of the innate immune system which is activated immediately upon pathogen detection and does not involve the development of antigenic immunological memory. Several receptors, including two proteins called natural killer proteins 46 (NKp46) and 44 (NKp44), are responsible for the activation of NK. It has been proven experimentally that only one protein of paramyxoviruses, namely HN, activates NK [71]. It is assumed that NK activation by a UV-inactivated Sendai virus [30] is caused by interaction between the HN protein and NKp46 and/or NKp44 receptors. Efficient binding of the HN protein to NKp46 and/or 44 NKp receptors results in the lysis of cells which have the HN protein or its fragments on their surface [72-74].

A study of a UV-inactivated Sendai virus showed that NK cells play an important role in the virus-mediated regression of tumor growth. In a mouse model of renal cancer, the anti-tumor effect of the virus was reduced when it was co-injected with an antibody against GM1 ganglioside, which reduced the number of NK cells [30].

*Induction of anti-tumor cytotoxic activity of T lymphocytes.* It has been shown that the Newcastle disease virus (NDV) enhances tumor-specific cytotoxic response of CD8 T-cells (CTLs) and increases the activity of T-helper CD4 cells in the absence of an antiviral T-cell response [73]. The UV-inactivated virus, which is unable to replicate, promotes the anti-tumor CTL-response as actively as intact NDV, which is capable of replication. Apparently, the effect of NDV on the CTL response is caused by the introduction of functional viral HN protein molecules into the membranes of tumor cells and stimulation of neuraminidase activity [73] (*Fig. 4*). Since Sendai virus HN proteins are highly homologous to NDV ones, the data suggest that a HN protein, regardless of its origin (Sendai virus, NDV or other related paramyxoviruses), activates both the responses associated with cytotoxic lymphocytes and the NK cells.

*Stimulation of dendritic cells*. Dendritic cells (DCs) are specialized antigen-presenting cells that can efficiently amplify both innate and acquired immune responses against various pathogens and tumors. Detection of a virus or other pathogens initiates a specific differentiation program in DCs, which makes them capable of activating naive T-cells.

**Fig. 5:**
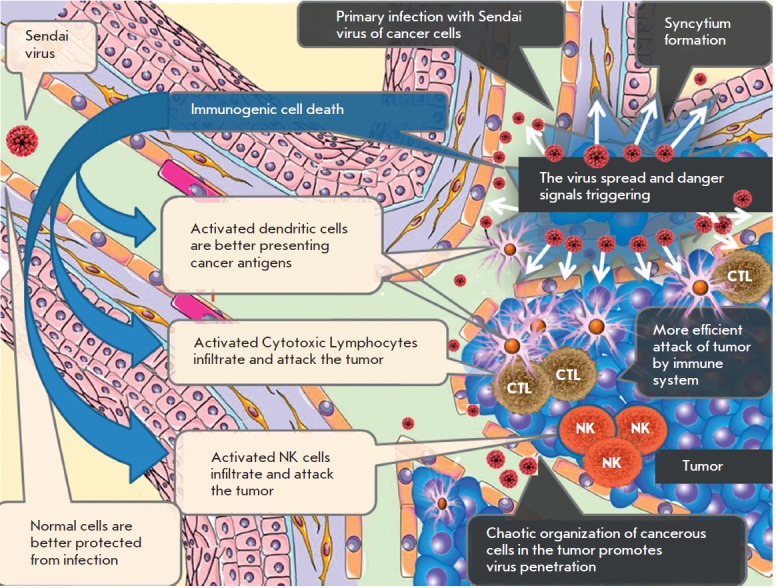
Sendai virus indices both direct and immune-mediated death of cancer cells. Cancer cells are more accessible to viruses and susceptible to viral replication. Ordered architecture of normal tissues (blood vessels, basal membranes, tight cell-to-cell contacts etc.) protects against viral invasion from the bloodstream. Chaotic organization of a tumor, loose cell-to-cell contacts, and leakiness of immature tumor vasculature provide better access to viruses. Normal cells exposed to viruses provide antiviral protection to surrounding normal cells by secreting IFNs. Tumor cells are generally defective for IFNs induction and, therefore, support viral replication even in the present of IFNs produced by the surrounding normal cells. The Sendai virus is capable of accelerated spread inside a tumor through the formation of syncytia. Exposure of viral antigens on the surface of infected cells induces massive immunogenic death of tumor cells. Virus-specific proteins represent danger signals triggering activation of innate and adaptive anticancer immune responses. Activated cytotoxic T lymphocytes (CTLs), natural killer (NKs), and antigen-presenting dendritic cells (DCs) migrate into the tumor and provide accelerated immune-mediated destruction of malignant cells

Even a UV-inactivated Sendai virus can cause intense infiltration of a tumor by dendritic cells [27], whereas *ex vivo *infection of DCs with a recombinant Sendai virus induces maturation and activation of DCs within an hour [74]. Administration of activated DCs carrying different variants of a recombinant Sendai virus significantly improves the survival of animals injected with malignant melanoma cells [75, 76], colorectal cancer [77], squamous cell carcinoma [78], hepatic cancer, neuroblastoma, and prostate cancer [26]. The use of such DCs prior to tumor cells administration has shown that DCs can prevent neuroblastoma and prostate adenocarcinoma metastasis into the lungs [79, 80]. The process of anti-tumor immunity activation by the Sendai virus is shown in *Fig. 5*.

*Suppression of regulatory cells. *Animal model experiments have shown that the Sendai virus is able to suppress T-cell-mediated regulatory immunosuppression by secretion of interleukin-6 by mature DCs even after UV inactivation [27].

## PROSPECTS OF FURTHER RESEARCH

Clinical trials of the Sendai virus are undoubtedly of interest. Currently, Japan is conducting phase I trials on the efficiency of a UV-irradiated virus in melanoma patients [81]. Its goal is to improve the systemic delivery of the inactivated virus to the tumor and metastases by pre-binding it to blood platelets. This approach has been tested on animals. It was found that binding to platelets significantly improves delivery of the virus and causes tumor growth suppression in murine melanoma models [82].

A study of a gene-engineered Sendai virus which can be activated by an altered spectrum of proteases is being conducted in Germany [83]. Animal experiments have shown that this virus can be easier to activate in malignant cells.

Another promising approach is the study of other oncolytic viruses whose co-administration with the Sendai virus could have a positive synergistic therapeutic effect.

## CONCLUSION

Several mechanisms explaining the oncolytic action of paramyxoviruses and, in particular, the Sendai virus have been established so far. The extent of the oncolysis and the specific mechanism of action may depend on several factors. Paramyxoviruses can directly kill cancer cells by multiplying within them and causing syncytium formation. The cells, which are fused into a syncytium, can no longer divide and are doomed to collective, synchronous death. Furthermore, paramyxoviruses induce immune-mediated killing of malignant cells via strong activation of anti-tumor NK cells, as well as via enhanced anti-tumor activity of cytotoxic T-cells, stimulation of antigen presenting dendritic cells, and immunosuppressive activity of suppressing T-cells. The neuraminidases of paramyxoviruses capsids can cleave sialic acids from the surface of malignant cells, unmasking tumor antigens present on the cell membrane. This renders cancer cells more visible to the immune system. Furthermore, viral neuraminidases can ensure strong specific affinity of the virus for sialic acid polymers, which are over-represented on cancerous cells' membranes. This increases the specificity of the virus in respect to primary tumor cells and metastases, but not normal cells. These mechanisms may substantiate antitumor activity of the Sendai virus detected in animals and humans. Therefore, there an objective rationale for further development of anticancer drugs based on paramyxoviruses and, in particular, on the Sendai virus.

## Abbreviations

NDV: Newcastle Disease Virus
MHC: Major Histocompatibility Complex
HN: Hemagglutinin Neuraminidase
DC: Dendritic Cells
IFN: Interferon
HPBL: Human Peripheral Blood Leucocyte
NA: Neuraminidase (sialidase)
NK: Natural Killer
CTL: Cytotoxic T-Cells
UV: Ultraviolet light
